# Insights into the Biomarker Potential of Humanin and Mots-c Expression and Telomere Length in Alzheimer’s Disease

**DOI:** 10.3390/ijms262210866

**Published:** 2025-11-09

**Authors:** Francisco Rodríguez-Esparragón, Sara E. Cazorla-Rivero, Eduardo Torrealba, Ángeles Cánovas-Molina, Ayose N. González-Hernández, Ruth Martín-Alfaro, María P. Afonso-Medina, María T. Martínez de Saavedra-Álvarez, Carmen G. Pérez-Santana, Carmen Bartolomé, Lidia Estupiñán, Jesús M. González-Martín, Bernardino Clavo

**Affiliations:** 1 Research Unit, Hospital Universitario de Gran Canaria Dr. Negrín, 35019 Las Palmas de Gran Canaria, Spain; scazorla@ull.edu.es (S.E.C.-R.); canovaspi@hotmail.com (Á.C.-M.); carmen.perez118@alu.ulpgc.es (C.G.P.-S.); cbardur@gobiernodecanarias.org (C.B.); lestqui@gobiernodecanarias.org (L.E.); josu.estadistica@gmail.com (J.M.G.-M.); bernardinoclavo@gmail.com (B.C.); 2 Fundación Canaria Instituto de Investigación Sanitaria de Canarias (FIISC), 35019 Las Palmas de Gran Canaria, Spain; angonherb@gobiernodecanarias.org (A.N.G.-H.); rmaralf@gobiernodecanarias.org (R.M.-A.); mafomedr@gobiernodecanarias.org (M.P.A.-M.); mmaralvf@gobiernodecanarias.org (M.T.M.d.S.-Á.); 3 Instituto Universitario de Enfermedades Tropicales y Salud Pública de Canarias, Universidad de La Laguna, 38296 La Laguna, Tenerife, Spain; 4 CIBER de Enfermedades Infecciosas, Instituto de Salud Carlos III, 28029 Madrid, Spain; 5 Faculty of Health Sciences, Middle Atlantic University (UNAM), 35017 Las Palmas, Spain; 6 Department of Neurology and Clinical Neurophysiology, Hospital Universitario de Gran Canaria Dr. Negrín, 35019 Las Palmas de Gran Canaria, Spain; 7 Department of Medical and Surgical Sciences, Universidad de Las Palmas de Gran Canaria, 35001 Las Palmas de Gran Canaria, Spain; 8 Chronic Pain Unit, Hospital Universitario de Gran Canaria Dr. Negrín, 35019 Las Palmas de Gran Canaria, Spain; 9 Faculty of Health Sciences, Universidad Fernando Pessoa Canarias, 35450 Las Palmas de Gran Canaria, Spain; 10 Department of Clinical Chemistry, Hospital Universitario de Gran Canaria Dr. Negrín, 35019 Las Palmas de Gran Canaria, Spain; 11 Department of Immunology, Hospital Universitario de Gran Canaria Dr. Negrín, 35019 Las Palmas de Gran Canaria, Spain; 12 Instituto Universitario de Sanidad Animal y Seguridad Alimentaria (IUSA), Universidad de Las Palmas de Gran Canaria (ULPGC), 35416 Las Palmas de Gran Canaria, Spain; 13 Radiation Oncology Department, Hospital Universitario de Gran Canaria Dr. Negrín, 35019 Las Palmas de Gran Canaria, Spain; 14 Molecular and Translational Pharmacology Group, University Institute for Research in Biomedicine and Health (iUIBS), Universidad de Las Palmas de Gran Canaria, 35016 Las Palmas de Gran Canaria, Spain; 15 Spanish Group of Clinical Research in Radiation Oncology (GICOR), 28290 Madrid, Spain

**Keywords:** Alzheimer’s disease, mild cognitive impairment, mitochondria, humanin, mitochondrial ORF of the 12S rRNA Type-C, biomarker, telomere length, gene expression

## Abstract

Humanin (HN) and MOTS-c are mitochondrial-derived peptides (MDPs) known for their neuroprotective and metabolic functions. Their circulating and tissue levels decline with age and in neurodegenerative diseases such as Alzheimer’s disease (AD). This study aimed to evaluate whether blood and plasma gene expression and plasma protein levels of HN and MOTS-c are associated with AD markers, their role in the conversion from mild cognitive impairment (MCI) to AD, and their overall association with the disease. A case–control study was conducted, including patients with AD and MCI, and individuals with subjective cognitive decline (SCD) as controls. Gene expression levels were quantified from total RNA isolated from blood and plasma, normalised to mitochondrial DNA copy number (mtDNA-CN). ELISA was used to measure plasma HN and MOTS-c protein concentrations. HN and MOTS-c transcript levels differed significantly among study groups, whereas plasma protein concentrations did not discriminate between AD and MCI. In silico and RNA decay assays revealed faster degradation of HN mRNA and delayed but stable recovery of MOTS-c mRNA. Overall, blood and plasma transcript levels—but not circulating protein levels—of these MDPs were significantly reduced in AD compared to SCD, suggesting their potential as early biomarkers of Alzheimer’s disease.

## 1. Introduction

Neurodegenerative diseases are among the leading causes of death and disability in the elderly population worldwide. Alzheimer’s disease (AD) is the most common neurodegenerative disorder [1], characterized by the deposition of β-amyloid (Aβ) plaques, neurofibrillary tangles (NFTs) formed by abnormally phosphorylated tau-protein, leading to massive neuronal cell death, severe cognitive deficits and progressive memory decline. Mitochondrial dysfunction is a well-established hallmark of AD. Alterations in mitochondrial bioenergetics, oxidative stress, and impaired mitophagy contribute to neuronal damage and cognitive deterioration. In recent years, mitochondrial-derived peptides (MDPs) such as Humanin (HN) and MOTS-c have gained attention as critical regulators of mitochondrial signaling, metabolism, and cellular protection, thereby linking mitochondrial health to neurodegenerative processes.

Humanin (HN) was first identified as an MDP capable of protecting neurons against Aβ-induced toxicity, a central event in AD pathogenesis [2,3]. Beyond its cytoprotective and anti-apoptotic actions, HN also exerts metalloprotective effects by binding transition metals such as copper (Cu^2+^) and zinc (Zn^2+^) [4,5], which are aberrantly accumulated in Aβ plaques [6]. These metal ions can catalyze the production of reactive oxygen species (ROS) and promote Aβ aggregation. By sequestering or modulating these redox-active metals, HN may reduce oxidative stress and inhibit metal-mediated neurotoxicity, thereby attenuating one of the major pathogenic pathways in AD. Humanin (HN) is a 24-amino-acid MDP encoded by the MT-*RNR2* gene, located within the 16S ribosomal RNA gene of the mitochondrial genome [2,3].

The Mitochondrial Open Reading Frame of the 12S ribosomal RNA-c (MOTS-c), another MDP encoded within the mitochondrial 12S rRNA, plays a complementary role in maintaining cellular homeostasis. It acts as a potent activator of the AMP-activated protein kinase (AMPK) pathway, thereby regulating glucose and lipid metabolism, enhancing mitochondrial biogenesis, and promoting antioxidant responses [7]. These mechanisms are particularly relevant in the brain, where energy imbalance and mitochondrial dysfunction contribute to neuronal vulnerability and cognitive decline. Moreover, MOTS-c has been reported to translocate to the nucleus under metabolic or oxidative stress, where it modulates gene expression involved in stress resistance and metabolic adaptation which are processes that are often impaired in AD [8]. MOTS-c is a 16-amino-acid MDP encoded by the *MT-RNR1* gene within the 12S ribosomal RNA gene of the mitochondrial genome [9].

Given their shared mitochondrial origin and overlapping protective functions [10], both HN and MOTS-c are of significant interest in the context of AD [11,12]. Previous studies have shown that circulating levels of these peptides decline with aging and metabolic disorders [10], but their role in AD progression and early stages such as mild cognitive impairment (MCI) remains poorly defined. Therefore, in this study, we investigated the expression levels of HN and MOTS-c in blood and plasma, as well as their circulating protein concentrations, to explore their association with AD-related biomarkers and cognitive status. We further examined whether these mitochondrial-derived peptides could serve as potential indicators of disease progression from MCI to AD.

## 2. Results

### 2.1. Patient’s Characteristics and Neurodegenerative CSF Markers Determination

A total of 169 participants were included in the study, comprising 88 patients with Alzheimer’s disease (AD), 36 individuals with mild cognitive impairment (MCI), and 45 participants with subjective cognitive decline (SCD), who served as controls. The mean age of the entire cohort was 73 ± 7.9 years, with no significant difference among groups (*p* = 0.11). Gender distribution was similar between AD and MCI patients, as indicated by the non-significant chi-square result (*p* = 0.76). However, gender distribution was significantly different between AD and subjective cognitive decline (SCD) participants (*p* = 0.02). The main characteristics of the studied patients, as well as the cerebrospinal fluid (CSF) levels of Aβ1–40, Aβ1–42, tau-protein and p-tau-181 levels are presented in Table 1. The table also includes mean pairwise comparisons between the studied groups.

### 2.2. Apolipoprotein E Genotyping

Apolipoprotein E (apoE) genotyping was performed on 154 of the 169 recruited patients. Among these, 13 individuals were identified as homozygous for apolipoprotein ε4 (apoE ε4), with 46% of them diagnosed with Alzheimer’s disease. Additionally, 61 patients were heterozygous carriers of the apolipoprotein ε4 allele, among whom 54% were diagnosed with Alzheimer’s disease (Table 2). Table 2 also presents the genotype and allele distribution for the entire cohort and across subgroups, along with Hardy–Weinberg equilibrium estimates for the three allele genotypes.

### 2.3. Blood Mitochondrial DNA Copy Number

**Figure 1:**
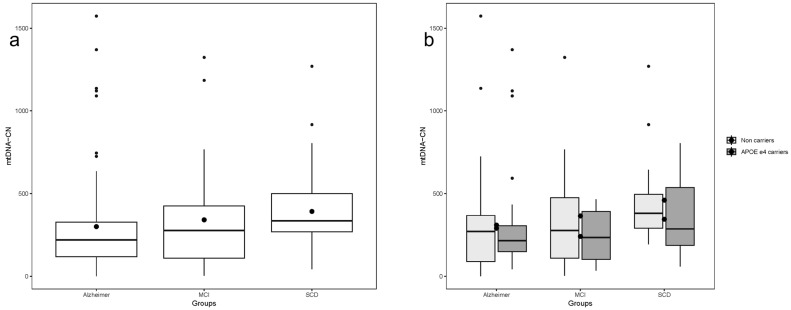
Mitochondrial DNA copy number (mtDNA-CN) by study group and APOE ε4 allele carrier status. (**a**) Mitochondrial DNA copy number (mtDNA-CN) differed significantly among study groups (*p* = 0.004). (**b**) When stratified by apoE ε4 allele carrier status, only non-carriers showed significant differences in mtDNA-CN between groups (*p* = 0.033). Data are shown as box plots, where boxes represent the interquartile range (IQR, Q1–Q3), the horizontal line inside each box indicates the median, whiskers denote the minimum and maximum values, and dots represent individual data points.

There was a significant difference in Mitochondrial DNA Copy Number (mtDNA-CN) values across patient classifications, with lower levels observed in AD patients and higher levels in SCD patients. (Kruskal–Wallis chi-squared = 10.669, df = 2, *p*-value = 0.004823) (Figure 1a). Pairwise comparisons between groups revealed a statistically significant difference in mtDNA-CN when comparing AD and SCD (Kruskal–Wallis chi-squared = 11.279, df = 1, *p*-value = 0.000783). Similarly, graded variation in mtDNA-CN values was observed across all participants when further divided by APOE ε4 allele carriers vs.. non-APOE ε4 carriers. However, the group comparison reached statistical significance only in non-carriers of the APOE ε4 allele (Kruskal–Wallis chi-squared = 6.7997, df = 2, *p*-value = 0.03338) (Figure 1b).

### 2.4. Humanin Transcript and Protein Levels in Blood and Plasma

Humanin (HN) transcript levels, normalized to blood mtDNA-CN, were assessed in whole blood and plasma samples. Whole blood HN expression significantly correlated with plasma HN expression (ρ = 0.346, *p* < 0.0001, *N* = 154), and with MOTS-c expression in blood (ρ = 0.7, *p* < 0.0001) and plasma (ρ = 0.196, *p* = 0.014). However, no correlation was found between HN transcript levels (blood or plasma) and plasma HN protein levels measured by ELISA. Plasma HN protein correlated negatively with age only in SCD patients (r = −0.47, *p* = 0.04)

**Figure 2:**
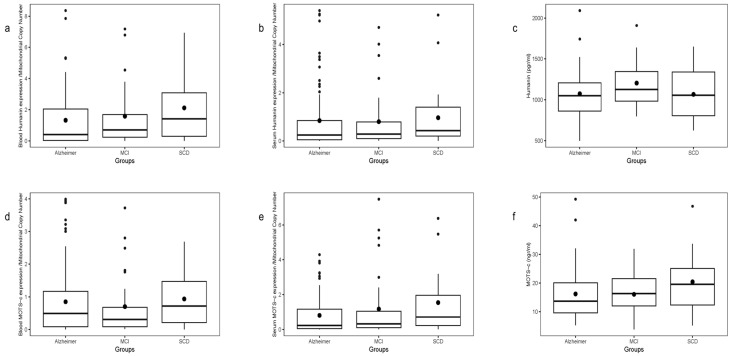
Blood and plasma transcript and protein levels of HN (**a**–**c**) and MOTS-c (**d**–**f**) across patient groups. HN and MOTS-c transcript and protein levels were measured in blood (**a**,**d**), plasma (**b**,**e**), and by ELISA (**c**,**f**) across patient groups. HN transcripts in blood were significantly higher in SCD than AD (*p* < 0,05), while other comparisons showed no significant differences. Box plots display the interquartile range (IQR), median (horizontal line), whiskers (minimum and maximum values), and dots (individual samples).

A gradient in mean HN transcript levels was observed across groups, lowest in AD, intermediate in MCI, and highest in SCD (Figure 2a). This difference was borderline overall (*p* = 0.0765), but significant in the AD vs. SCD two-group comparison (*p* = 0.0198). Plasma HN transcript levels showed a similar trend without significant differences (Figure 2b), and plasma HN protein levels measured by ELISA showed no differences among groups or in pairs (Figure 2c).

### 2.5. Stratification of Humanin Expression by APOE ε4 Status

**Figure 3:**
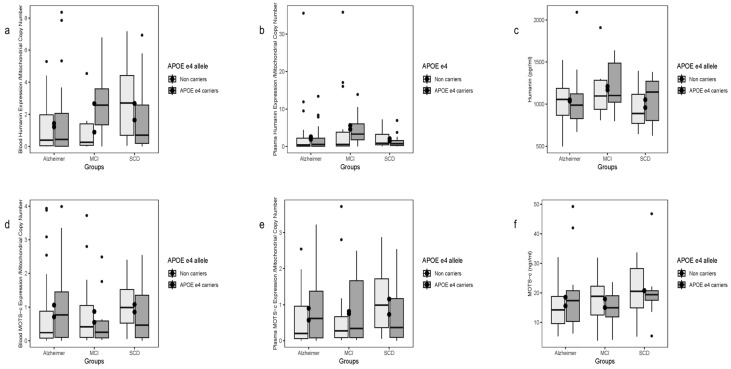
Blood and plasma transcript and protein levels of HN (**a**–**c**) and MOTS-c (**d**–**f**) across patient groups and apoE status. (**a**) Blood HN transcripts differed significantly among groups in non-apoE ε4 carriers, with significant contrasts between AD vs. SCD and MCI vs. SCD. (**b**–**c**) Plasma HN transcripts and protein levels showed no significant differences by APOE ε4 status. (**d**–**f**) MOTS-c transcript and protein levels in blood, plasma, and determined by ELISA showed similar trends across groups, without statistically significant differences. Data are shown as box plots: boxes indicate the IQR (Q1–Q3), horizontal lines represent the median, whiskers denote the minimum and maximum, and dots correspond to individual data points.

Blood HN transcript levels differed significantly by APOE ε4 status (*p* = 0.0465), driven by non-carriers, where two-group contrasts AD vs. SCD and MCI vs. SCD were significant (Figure 3a). No differences were seen for plasma HN transcripts or plasma protein levels by APOE ε4 status (Figure 3b,c). Plasma transcript and protein HN levels showed no overall correlations.

### 2.6. MOTS-c Transcript and Protein Levels in Blood and Plasma

Blood MOTS-c transcript levels normalized to mtDNA-CN did not differ by patient group and showed no correlation with plasma MOTS-c protein levels measured by ELISA (Figure 2d). No significant age correlation was observed for plasma MOTS-c protein.

Plasma MOTS-c transcript levels exhibited a slight gradient (lowest in AD, highest in SCD), with a significant difference in AD vs. SCD two-group analysis (Figure 2e). No correlation was found between transcript levels and plasma protein concentrations. Plasma MOTS-c protein levels did not vary significantly by group (Figure 2f).

### 2.7. Stratification of MOTS-c Expression by APOE ε4 Status

No differences were observed in blood or plasma MOTS-c transcript levels or plasma protein concentrations when stratified by disease or APOE ε4 carrier status (Figure 3d–f).

### 2.8. Evaluation of HN and MOTS-c Expression in GSE282742

**Figure 4:**
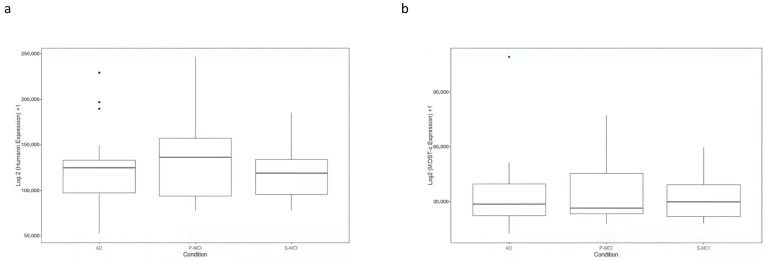
(**a**) Expression levels of HN and MOTS-c across AD, progressive MCI (P-MCI), and stable MCI (S-MCI) groups in GSE282742. Boxplots show log2-transformed counts per million (log2(CPM + 1)) for HN (**a**) and MOTS-c transcripts (**b**). Mean HN expression was lower in AD compared to P-MCI, while MOTS-c levels were similar across groups. No statistically significant differences were observed. Boxes represent the IQR; horizontal lines the median, whiskers the minimum and maximum, and dots individual samples.

The expression data for HN and MOTS-c in GSE282742 are shown in Figure 4. The plot displays log2-transformed CPM values (log2 (CPM+1) for each gene. The mean expression levels of HN appeared lower in AD compared to P-MCI, while similar levels were observed between AD and S-MCI. However, no statistically significant differences were found among all groups analysed or in any pairwise comparisons. Likewise, no significant differences in MOTS-c expression were observed.

### 2.9. Telomere Length

**Figure 5:**
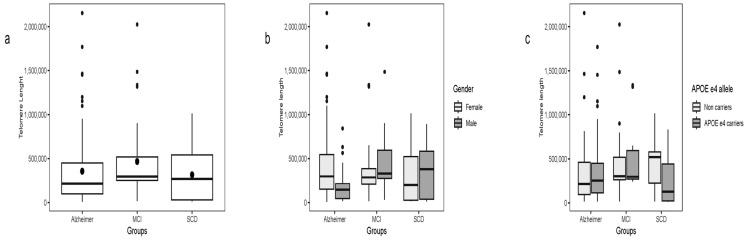
Telomere length across patient subgroups by diagnosis, gender, and apoE ε4 status. Boxplots show telomere length in diagnostic groups: Alzheimer’s disease (AD), mild cognitive impairment (MCI), and subjective cognitive decline (SCD) (**a**); stratified by gender within each group (**b**); and stratified by APOE ε4 carrier status within each group (**c**). Boxes represent the interquartile range (IQR), horizontal lines indicate the median, whiskers show the minimum and maximum values, and dots represent individual data points.

Telomere length was evaluated across all groups without overall differences. A marginally significant difference was observed when comparing AD patients with MCI (χ^2^ = 3.84, df = 1, *p* = 0.04), while no other pairwise differences were detected (Figure 5a).

Across the full cohort, telomere length negatively correlated with HN transcript levels in whole blood (ρ = −0.35, *p* < 0.001) and plasma (ρ = −0.24, *p* = 0.002). These associations remained significant in separate analyses of AD and SCD patients. No correlation was detected between telomere length and plasma HN protein concentration.

Similarly, whole blood MOTS-c transcript levels were negatively associated with telomere length (ρ = −0.30, *p* < 0.001). The association for plasma MOTS-c transcript levels was marginal (ρ = −0.14, *p* = 0.06). Plasma MOTS-c protein levels showed no correlation with telomere length.

Subgroup analyses revealed significant negative correlations between whole blood MOTS-c transcripts and telomere length in AD (ρ = −0.30, *p* = 0.005) and SCD patients (ρ = −0.40, *p* = 0.007). In the SCD subgroup, plasma MOTS-c transcripts also correlated with telomere length (ρ = −0.49, *p* < 0.001), whereas plasma protein levels remained unrelated.

When telomere length was compared within subgroups stratified by gender and APOE ε4 status (Figure 5c,d), no significant differences were observed. The exception was within the SCD group, where APOE ε4 carriers showed shorter telomeres (χ^2^ = 4.71, df = 1, *p* = 0.03).

### 2.10. In Silico RNA Stability Analysis

The MFE values for the RNA sequences were −12.3 kcal/mol for HN and −10.8 kcal/mol for MOTS-c RNA. These results suggest that both RNAs form stable and energetically favorable secondary structures.

### 2.11. Protein Stability Metrics

The stability analysis of the corresponding proteins revealed that the HN protein Instability Index was 45.2, which classified it as unstable. (Molecular Weight = 2.37 kDa and pI = 10.1) whereas for the MOTS-c protein, the Instability Index was 38.7, which classified it as stable. (Molecular Weight = 2.44 kDa and pI = 9.8). The estimated half-lives of the proteins were 1.1 h for HN and 4.4 h for MOTS-c. Thus, HN protein has a shorter half-life and is less stable compared to MOTS-c.

### 2.12. Normalized Data and Comparative Visualization

**Figure 6:**
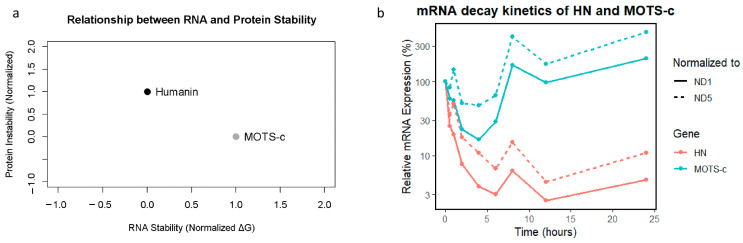
Correlation of predicted RNA stability and protein instability for HN and MOTS-c, and mRNA decay kinetics in HeLa cells. (**a**) Scatter plot showing the relationship between predicted RNA stability (Minimum Free Energy, MFE; calculated by RNAfold) and protein instability index (calculated by ProtParam/UniProt) for Humanin (HN) and MOTS-c. Each point represents the values for one peptide. HN RNA shows higher predicted stability (more negative MFE) but codes for a less stable protein (higher instability index and shorter half-life), while MOTS-c RNA is less stable but codes for a more stable protein. (**b**) mRNA decay kinetics in HeLa cells following transcriptional inhibition. HN degrades rapidly with limited recovery, whereas MOTS-c displays delayed but pronounced rebound in mRNA levels, particularly when normalized to ND5.

All metrics were normalized and plotted to facilitate a cohesive comparison: RNA stability (normalized MFE) showed consistent and high stability. Proteins exhibited greater variability, particularly in half-life and instability index. The composite graph visually emphasizes the stability of RNA relative to protein instability, supporting the notion that RNA molecules are inherently more resilient (Figure 6a).

### 2.13. HN and MOTS-c RNA Decay

To assess the post-transcriptional dynamics of HN and MOTS-c, we performed an mRNA decay assay in HeLa cells after transcriptional inhibition. The results revealed that HN mRNA levels normalized to both ND1 and ND5 gene amplicons decreased rapidly, with expression dropping below 5% within 6 h and remaining low throughout the 24-h time course. In contrast, *MOTS-c* mRNA initially decreased modestly but exhibited a striking rebound after 6–8 h and continued rising at 24 h. This divergent behavior suggests a fundamental difference in transcript stability and/or regulatory control between the two genes. These findings are illustrated in Figure 6b, which depicts the mRNA decay kinetics over time.

### 2.14. ROC Analysis Results of HN and MOTS-c

**Figure 7:**
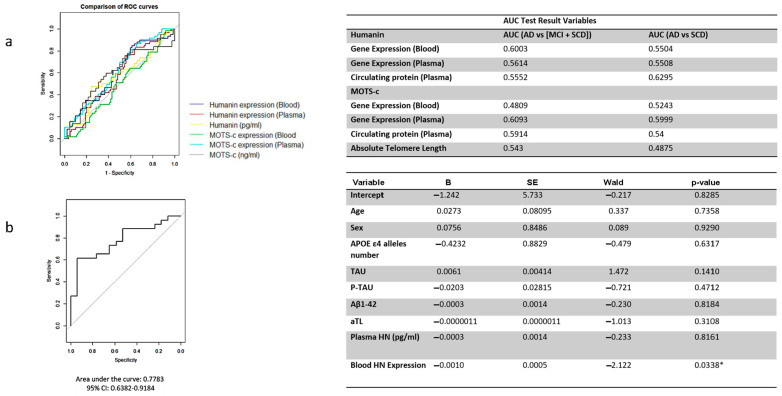
ROC analysis and logistic regression summary for HN and MOTS-c peptides. (**a**) ROC curve analysis illustrating the diagnostic performance of Humanin (HN) and MOTS-c for distinguishing Alzheimer’s disease (AD) from combined mild cognitive impairment (MCI) and subjective cognitive decline (SCD) groups, and from SCD alone. Curves show sensitivity versus specificity, with the corresponding area under the curve (AUC) values displayed on the right. (**b**) ROC curve derived from the binary logistic regression model (left) and summary table (right) presenting regression coefficients (B), standard errors (SE), Wald statistics, and *p*-values for variables included in the model. The AUC values in (**b**) reflect the internal discriminative capacity of the logistic model.

The diagnostic performance of HN and MOTS-c gene expression, measured in whole blood and plasma, as well as the circulating plasma levels of these peptides, was insufficient for distinguishing AD from MCI and SCD patients considered together. It was also inadequate for differentiating AD from individuals with SCD (Figure 7a).

### 2.15. Logistic Regression Analysis

Although individual ROC analyses for HN, MOTS-c, and telomere repeat markers did not demonstrate strong discriminative power when considered separately, a multivariable logistic regression model combining blood HN expression, MOTS-c expression, telomere repeats, and other relevant covariates provided greater diagnostic insight. The ROC curve derived from the model’s predicted probabilities showed improved performance, with an AUC of 0.78, indicating its potential utility in distinguishing between cognitive states.

Among the variables included, only blood HN expression emerged as a statistically significant predictor of cognitive status, as shown in Figure 7b. This suggests that HN levels in peripheral blood may independently contribute to the differentiation of clinical groups, while the combined model underscores the importance of integrated biomarker approaches.

## 3. Discussion

Mitochondrial-derived peptides (MDPs) are a novel class of bioactive microproteins encoded by short open-reading frames (sORFs) within known mitochondrial DNA genes. To date, eight MDPs have been identified. Among them, Humanin (HN, *MT-RNR2*) is a 24-amino acid MDP that is encoded within the 16S ribosomal RNA gene [2,3], and MOTS-c (Mitochondrial ORF within 12S ribosomal RNA type-c, *MT-RNR1*) is a 16-amino acid MDP encoded within the 12S ribosomal RNA gene [11]. Both HN and MOTS-c play an essential role in neuroprotection, modulation of cellular metabolism, and cytoprotection [10,11]. However, despite their shared functional similarities, these MDPs have distinctive physiological and pathophysiological functions [10].

HN has been shown to protect neurons from neurotoxicity-induced cell death through mechanisms such as binding to the insulin-like growth factor binding protein-3 (IGFBP3), thereby blocking IGFBP3-induced cell apoptosis. Additionally, HN inhibits apoptosis by interacting with the Bcl-2 family protein Bax [13]. It also mediates various metabolic effects via its binding to several surface receptors [11,14,15]. In contrast, no specific receptors for MOTS-c have been identified [9]. Instead, MOTS-c translocates to the nucleus through an AMPK-dependent mechanism and regulates transcription factors such as NRF1 (Nuclear Respiratory Factor 1) and NRF2 (Nuclear Factor, Erythroid 2 Like 2), promoting cellular homeostasis [12]. Moreover, MOTS-c regulates energy metabolism, ameliorates insulin resistance, and reduces pro-inflammatory cytokines via activation of the AMP-activated protein kinase and inhibition of mitogen-activated protein kinase/nuclear factor kappa-light-chain-enhancer of activated B (MAP/NF-κB) [16].

In this study, we investigated whether circulating transcript and plasma protein levels of HN and MOTS-c could serve as biomarkers for Alzheimer’s disease (AD) risk and progression across the cognitive impairment spectrum. Studies have shown that mitochondrial DNA copy number (mtDNA-CN) is itself a marker of neurodegeneration [17], increased risk of conversion from MCI to AD and of mitochondrial dysfunction in AD [18]. To control for inter-individual mitochondrial variability, we normalized HN and MOTS-c transcripts levels to blood mtDNA-CN. Normalized blood transcript levels of HN and MOTS-c showed gradual relationships according to disease states, with the lowest levels in AD patients, slightly higher levels in MCI patients, and the highest levels in controls with subjective cognitive decline (SCD). Statistically significant differences were observed between AD patients and SCD controls. These findings support the hypothesis that MDPs may act as early indicators of mitochondrial dysfunction in neurodegeneration. However, protein levels of HN and MOTS-c in plasma did not show significant differences across groups. This discrepancy may arise from post-transcriptional regulatory mechanisms, including mitochondrial translation control and differential peptide degradation rates in circulation. Additionally, while transcript levels reflect gene expression, plasma protein concentrations depend heavily on peptide stability and clearance dynamics [19,20]

Previous studies have reported lower HN protein levels in patients with AD compared to controls. Notably, HN protein levels in cerebrospinal fluid (CSF) were significantly reduced in AD patients [21,22], suggesting that diminished levels of this mitochondrial peptide may contribute to cognitive decline. Additionally, a study comparing HN levels in patients with type 2 diabetes (T2D) and AD reported reduced HN protein levels in AD but not in T2D [23]. In contrast, MOTS-c has not been sufficiently studied in AD, but its role in protecting cells against oxidative stress and aging suggests that it might be involved in AD pathogenesis.

An important observation in our study is that, while the predicted MFE (Minimum Free Energy) prediction suggests stable secondary RNA structures for HN and MOTS-c transcripts, our experimental mRNA decay assays in HeLa cells reveal a different picture. HN mRNA, despite its lower (more negative) MFE, underwent rapid degradation with no recovery over 24 h. Conversely, MOTS-c mRNA, with a slightly less negative MFE, exhibits delayed but robust reaccumulation between 6 and 24 h. This suggests that structural RNA stability alone does not predict functional RNA persistence, underscoring the importance of additional regulatory mechanisms, such as RNA-binding proteins or feedback transcriptional control.

At the protein level, the observed difference in stability remains consistent: HN has a short half-life and is classified as unstable, supporting a role as a fast-acting, transient stress-responsive peptide. In contrast, MOTS-c exhibits a longer half-life and greater protein stability, consistent with its role in sustaining metabolic regulation and cellular adaptation. Together, these data suggest that HN and MOTS-c operate on distinct temporal scales. Thus, HN response is rapid and transient, whereas MOTS-c seems to act over longer periods, possibly to maintain homeostasis during prolonged stress.

Analysis of publicly available transcriptomics data (GSE282742) provides no additional findings. HN transcripts showed measurable expression across cognitive states and broadly mirrored patterns observed in our cohort. The intermediate levels observed in the P-MCI group suggest its transitional nature, where early molecular changes may begin to emerge. However, no statistically significant differences were detected between groups. MOTS-c transcripts were also present in peripheral blood mononuclear cells (PBMCs) but did not exhibit expression differences across cognitive states. While these observations do not support the use of HN or MOTS-c expression levels as standalone biomarkers, they do reinforce the idea that both peptides are consistently expressed in peripheral blood and may still participate in early regulatory responses during cognitive decline. The rapid decay observed for *HN* mRNA in our experimental data may also suggest a tightly regulated expression profile, making it a dynamic rather than stable marker, potentially more informative when combined with other time-sensitive molecular readouts

Telomere length (TL) is a marker of genome stability and cellular aging, with shorter TL associated with inflammation and oxidative stress [24]. Although telomere shortening contributes to cellular aging in the central nervous system, studies on TL in AD have shown non-linear associations [25]. Furthermore, leukocyte telomere length (LTL) has been linked to cognitive ability [26]. Our study revealed a negative correlation between LTL and levels of HN and MOTS-c, suggesting that as telomeres shorten, levels of these MDPs decrease. This relationship may reflect a decline in cellular resilience and mitochondrial health associated with aging and neurodegeneration. However, we did not observe significant differences in telomere length across the disease groups, suggesting that the relationship between telomeres and MDPs could be more complex and would require a longitudinal analysis to be validated. This negative correlation aligns with research indicating bidirectional interactions between mitochondrial function and telomere biology during cellular aging. Mitochondrial dysfunction can accelerate telomere shortening, while telomere attrition may impair mitochondrial biogenesis and function, together influencing cellular senescence [21,27,28].

Mitochondrial dysfunction is a recognized hallmark of AD, involving impaired energy production, increased reactive oxygen species (ROS), and defective mitophagy. Given that MDPs, including HN and MOTS-c, are protective peptides produced in response to mitochondrial stress, their decreased transcript levels might reflect early mitochondrial dysregulation in AD. Thus, circulating transcript levels of HN and MOTS-c might hold greater potential as biomarkers than the protein levels themselves [27,29,30].

Notwithstanding the above, we have several important limitations in our study. We have a limited sample size, and we also observed a poor discriminatory power of HN and MOTS-c levels as biomarkers for AD based on ROC curve analysis; therefore, these MDPs alone might not serve as effective standalone biomarkers for disease diagnosis. Moreover, our results indicate that MDPs are associated with cellular aging markers but may have limited diagnostic utility. Nevertheless, as multivariate models suggest, the integration of MDP transcript levels with established biomarkers such as amyloid-β and tau could enhance the diagnostic accuracy for AD. This combinatory approach may also help delineate early mitochondrial dysfunction from downstream neurodegenerative processes. Future research should explore their role in combination with other markers or focus on their therapeutic potential to support cellular resilience in aging and neurodegeneration. Other potential limitations include inter-individual variability in transcript and protein levels, differences in quantification methodologies, and the need for longitudinal analyses to validate the predictive value of MDPs in AD progression. It is important to consider age and gender as confounding variables that influence the expression of mitochondrial-derived peptides. Recent studies report age- and sex-specific differences in mitochondrial quality control that could affect MDP levels [31] and their interpretation in neurodegenerative contexts, highlighting the need to control for these factors in future studies [21]. Addressing inter-individual variability and improving quantification techniques through advanced transcriptomics could enhance the reliability of MDPs as biomarkers. Longitudinal studies are critical to establish whether MDP reductions precede or accompany AD onset.

In conclusion, despite study limitations, our findings suggest that HN and MOTS-c transcripts may serve as early, dynamic markers of mitochondrial dysfunction in AD. Integrating these markers with established AD biomarkers could improve diagnostic accuracy and enable earlier interventions. The therapeutic modulation and/or modifications of MDPs represent a promising avenue to bolster mitochondrial resilience and slow neurodegeneration.

## 4. Materials and Methods

### 4.1. Patient Recruitment and Diagnostic Criteria

A total of 124 patients were recruited from the Neurology Department of Hospital Universitario de Gran Canaria Dr. Negrín (HUGCDN) and classified according to clinical diagnosis as Alzheimer’s disease (AD; *n* = 88) or mild cognitive impairment (MCI; *n* = 36). In addition, forty-five older individuals with subjective memory complaints but no objective cognitive impairment were included as controls with subjective cognitive decline (SCD).

All participants were evaluated by experienced neurologists following a standardized diagnostic protocol that included medical and family history, neurological examination, and comprehensive neuropsychological testing. Global cognition was assessed using the Mini-Mental State Examination (MMSE), and a detailed neuropsychological battery adapted from the Development of Screening Guidelines and Criteria for Predementia Alzheimer’s Disease (DESCRIPA) study was administered to assess memory, language, praxis, visuospatial abilities, and executive function. Tests included the Spanish version of the Free and Cued Selective Reminding Test (FCSRT), the Rey–Osterrieth Complex Figure, a short version of the Boston Naming Test, semantic fluency (animals category), the Token Test, the Stroop Color–Word Test, and WAIS subtests (Digit Span and Digit Symbol). Functional status was evaluated by the Blessed Dementia Rating Scale (BDRS) and Instrumental Activities of Daily Living (IADL), global deterioration by the Global Deterioration Scale (GDS), and mood with the Hospital Anxiety and Depression Scale (HADS).

Probable AD diagnosis was established according to the NINCDS–ADRDA and DSM-IV-TR criteria, integrating clinical, neuropsychological, and functional data (BDRS part A > 1.5 and IADL < 6 for women and < 5 for men). MCI diagnosis followed the European Consortium on Alzheimer’s disease (EADC) criteria, requiring objective impairment (>1.5 SD below age- and education-adjusted norms) in at least one cognitive domain, preserved general cognitive function, and intact daily activities.

SCD was defined as the presence of self-reported cognitive complaints in the absence of measurable impairment on the neuropsychological battery or functional decline in daily life.

Neuroimaging (MRI or CT), electroencephalogram (EEG), and cerebrospinal fluid (CSF) biomarkers (Aβ1–42, total tau, and phosphorylated tau [p-tau_181_p]; INNOTEST ELISA, Fujirebio, Ghent, Belgium) were used as supportive diagnostic tools when available.

The progressive MCI (P-MCI) and stable MCI (S-MCI) categories mentioned in this study refer exclusively to the external transcriptomic dataset GSE282742 used for in-silico analyses and were not applied to the present clinical cohort.

The study was approved by the Ethics Committee of HUGCDN (Reference Number: 2020-277-1) and conducted according to the Declaration of Helsinki. All participants provided written informed consent.

### 4.2. Sample Collection, MDPs Gene Expression and ELISA Measurements

Blood samples were collected from all patients and control subjects in RNase-free tubes containing EDTA. To isolate plasma, 3 mL of whole blood was centrifuged at 4 °C. Aliquots of whole blood and plasma were then stored at −80 °C until further use. Before RNA extraction, blood samples diluted in molecular biology–grade, RNase-free phosphate-buffered saline (PBS; Sigma-Aldrich, St. Louis, MO, USA). PBS and undiluted plasma samples were thawed.

Total RNA was extracted using Trizol reagent (Invitrogen, Carlsbad, CA, USA) according to the manufacturer’s protocol, and cDNA was synthesized with the iScript kit (Bio-Rad, Herncules, CA, USA). Humanin (HN) gene expression was analyzed in whole blood and plasma samples via real-time PCR, using previously described primers while Mitochondrial Open Reading Frame Of The 12S rRNA-c (MOTS-c) expression was quantified using the primers 5′-AGGTGGCAAGAAATGGGCTA-3′ and 5′-ATACTTGAGGAGGGTGACGG-3′. Amplifications were performed in a Bio-Rad CFX96 C1000 thermocycler using SYBR Green fluorescence detection chemistry. Relative expression levels were calculated using the comparative ΔΔCt method adapted for haploid mitochondrial genes, normalizing transcript abundance to blood mitochondrial DNA copy number (mtDNA-CN). All amplifications except APOE genotyping were done in a Biorad CFX96 C1000 device.

Circulating levels HN and MOTS-c were measured in the plasma of patients and controls using ELISA kits. The MT-RNR2 ELISA kit (CUSABIO, Wuhan, China; detection range: 28–1800 pg/mL; sensitivity 7 pg/mL) and the MOTS-c ELISA kit (LOUD-CLONE CORP., USA; detection range: 0.156–10 ng/mL, minimum detectable dose: < 0.057 ng/mL), were used following the manufacturers’ instructions.

Cerebrospinal fluid (CSF) core Alzheimer’s disease biomarkers (Aβ1–42, Aβ1–40, total tau, and phospho-tau 181) were quantified at the Clinical Analysis Service of the Hospital Universitario de Gran Canaria Dr. Negrín (HUGCDN) using a fully automated chemiluminescent enzyme immunoassay system (CLEIA; Lumipulse^®^ G600II, Fujirebio, Tokyo, Japan). Calibration and quality control procedures were performed according to the manufacturer’s instructions. The analytical quantification ranges were approximately 100–3,500 pg/mL for Aβ1–42, 200–15,000 pg/mL for Aβ1–40, and 50–2250 pg/mL for total tau. The laboratory’s validated cut-off points were applied, and results were considered abnormal when Aβ1–42  <  500 pg/mL and phospho-tau181  >  80 pg/mL.

### 4.3. Blood Mitochondria DNA Copy Number (mtDNA-CN)

Genomic DNA was extracted from blood samples by Phenol-Chloroform procedure and stored in pure water at −20 °C at a concentration of 100 ng/mL. DNA stocks were diluted into pure water just prior to setting up real-time quantitative PCR (qPCR) runs. MtDNA-CN was determined as described [32].

### 4.4. APOE Genotyping

APOE gene polymorphism was determined using a commercial kit (TIB MOLBIOL LightMix^®^Kit APOE C112R R158C) (TIB MOLBIOL GmbH, Berlin, Germany) with the LightCycler^®^ FastStart DNA Master HybProbe in a Roche Light Cycler 1.5 apparatus (Roche Diagnostics, Mannheim, Germany).

### 4.5. Expression Levels in a GEO Database

Comparison with published data was conducted evaluating the expression levels of HN and MOTS-c across different cognitive states using the publicly available dataset GSE282742, which includes transcriptomic data from Peripheral Blood Mononuclear Cells (PBMCs) of individuals with Alzheimer’s disease (AD, n = 49), Progressive Mild Cognitive Impairment (P-MCI, n = 28), and Stable MCI (S-MCI, n = 49).

The dataset was preprocessed to remove inconsistent or incomplete samples. Differential gene expression analysis was performed using DESeq2, which operates on raw counts to identify significant expression changes in HN and MOTS-c across groups.

Since no statistically significant differences were observed, we further examined raw counts without filtering for low-expression genes. These counts were then transformed to counts per million (CPM) and normalized using the edgeR package(version 3.40.2) [33] to enhance interpretability and reduce skewness. The normalized values were then log2-transformed (log2 (CPM + 1)) allowing for clearer visual exploration of expression trends across groups.

### 4.6. Absolute Telomere Length Quantification

Absolute Telomere Length (aTL) were measured in diluted leukocyte extracted DNA samples of all studied patients as previously described [34,35]. A standard curve of known quantities of a synthetic 84 mer oligonucleotide containing only TTAGGG repeats was used in aTL determinations.

### 4.7. In Silico Analysis of Humanin and MOTS-c RNA and Protein Stability

To evaluate HN and MOTS-c RNA stability, the minimum free energy (MFE) for the RNA sequences was calculated using RNAfold available from http://rna.tbi.univie.ac.at/cgi-bin/RNAWebSuite/RNAfold.cgi (accessed on 13 December 2024). MFE is a thermodynamic measure of RNA stability, with more negative values indicating more stable RNA secondary structures.

Protein Stability was assessed using the ProtParam available from https://web.expasy.org/protparam/ (accessed on 13 December 2024). HN and MOTS-c proteins were analyzed with ProtParam, focusing on three key stability-related metrics including the Instability index, which classifies proteins as stable or unstable and isoelectric point (pI), which provides additional insights into protein properties. In addition, the estimated half-lives of the proteins were calculated based on the N-terminal amino acid of the protein sequence and general degradation rules for mammalian cells.

To compare RNA and protein metrics, all values (MFE, instability index, molecular weight, and pI) were normalized using min-max normalization. The normalized values, ranging between 0 and 1, were visualized in a composite graph for direct comparison.

### 4.8. HN and MOTS-c RNA Decay Evaluation

To assess the post-transcriptional stability of HN and MOTS-c RNAs, an RNA decay assay was performed in HeLa cells following transcriptional arrest induced by ethidium bromide, as described [36,37].

### 4.9. Statistical Analysis

Statistical analyses were performed using R and RStudio for Windows (versions 4.3.1 and 2023.09.0). Numerical variables were expressed as mean ± standard deviation, while categorical variables were presented as counts and percentages. The normality of continuous variables was assessed using the Shapiro–Wilk test. Baseline data for age and categorical variables were compared between patients with AD, MCI, and SCD controls using the Kruskal–Wallis test and chi-square test, respectively. For continuous variables, comparisons between groups were made using one-way analysis of variance (ANOVA) for normally distributed parameters and the Kruskal–Wallis test for non-normally distributed parameters. Correlation analyses were conducted to evaluate the impact of gene expression on the analyzed groups.

Hardy–Weinberg equilibrium estimates for the APOE three alleles genotypes were calculated using the “HardyWeinberg” R package(version 1.7.9) [38].

Receiver Operating Characteristics (ROC) curve analyses were used to compare the gene expression levels between two groups (AD vs. (MCI + SCD) and AD vs. SCD. The area under the ROC curve, ROC test and probability value, cut point, establishing numerical measurements of sensitivity and specificity for the cut point, and generating the ROC curve graph are all part of these analyses.

Logistic regression models were constructed to differentiate AD patients from MCI and SCD controls combined, as well as to distinguish AD from SCD alone. To assess the performance of these models, ROC curve analyses were performed using the pROC package (version 1.18.5). Model fit was evaluated using the Hosmer–Lemeshow goodness-of-fit test, and the proportion of variance explained by the final model was determined using the Nagelkerke R^2^ statistic.

The statistical significance level was set at *p* < 0.05 for all analyses.

## Figures and Tables

**Table 1:** Biochemical parameters across study groups.

	ALL	AD	MCI	SCD	AD vs. MCI *p*-Value	AD vs. SCD *p*-Value	MCI vs. SCD *p*-Value
Variable							
Aβ1–40 (pg/mL)							
Mean ± SD	1239 ± 4068	14627 ± 3680	10088 ± 3637	9927 ± 1551	0.005	<0.001	0.900
Median (IqR)	11310 (9672–15270)	14768 (12391–16371)	9780 (8182–10645)	9980 (9566–11310)			
Aβ1–42 (pg/mL)							
Mean ± SD	697 ± 390	647 ± 355	631 ± 286	913 ± 508	0.838	0.039	0.037
Median (IqR)	617 (492–829)	547 (425–716)	550 (413–760)	897 (647–1065)			
Tau (pg/mL)							
Mean ± SD	513 ± 285	558 ± 265	440 ± 251	458 ± 355	0.084	0.261	0.853
Median (IqR)	497 (261–661)	554 (336–687)	441 (211–560)	352 (284–516)			
Tau_181p_ (pg/mL)							
Mean ± SD	68 ± 33	73 ± 30	69 ± 41	53 ± 26	0.689	0.009	0.158
Median (IqR)	63 (41–82)	69 (51–89)	62 (32–87)	50 (37–61)			
Glycemia (mg/mL)							
Mean ± SD	104.7 ± 27.9	114.7 ± 37.2	113.4 ± 28.8	106.6 ± 22.0	0.846	0.145	0.279
Median (IqR)	98.0 (65–108)	103.0 (94.5–119)	105.0 (94.0–115)	99.5 (91.0–115)			
HbA1c (%)							
Mean ± SD	6.08 ± 0.96	6.1 ± 1.1	6.2 ± 1.0	5.8 ± 0.6	0.542	0.135	0.081
Median (IqR)	5.8 (4.4–6.3)	5.8 (5.5–6.2)	5.8 (5.5–6.9)	5.6 (5.4–6.0)			
Cholesterol (mg/dL)							
Mean ± SD	197.8 ± 40.05	187.2 ± 37.2	193 ± 36.8	186.4 ± 43.5	0.455	0.918	0.498
Median (IqR)	195 (91–228)	186 (159–208)	184.0 (166–208)	182.0 (149–209.5)			
HDL-chol. (mg/dL)							
Mean ± SD	53 ± 17.4	59.5 ± 16.4	64.0 ± 28.1	56.6 ± 16.5	0.403	0.381	0.196
Median (IqR)	50 (21–62)	58.5 (47.7–69.7)	53 (45.5–74)	56 (46.2–65)			
LDL-chol. (mg/dL)							
Mean ± SD	120.8 ± 36.2	106.4 ± 28.4	107.8 ± 34.2	113.4 ± 40.1	0.843	0.422	0.587
Median (IqR)	115 (50–144)	103 (85.7–187)	97.0 (84–134)	104 (85–127.5)			
Urea (mg/dL)							
Mean ± SD	26.4 ± 19.3	36.2 ± 15.1	39.5 ± 9.8	11.8 ± 16.6	0.424	<0.001	<0.001
Median (IqR)	30.0 (1.1–36.7)	31.0 (27.5–38)	37.0 (32–49)	1.05 (0.8–28)			
Creatinine (mg/dL)							
Mean ± SD	1.6 ± 4.9	0.93 ± 0.28	3.3 ± 13.4	4.9 ± 5.2	0.327	<0.001	0.529
Median (IqR)	0.87 (0.49–1)	0.87 (0.73–1.04)	0.89 (0.78–0.95)	2.2 (0.9–7.1)			
Folic acid (ng/mL)							
Mean ± SD	65.11 ± 172.2	7.7 ± 4.2	7.9 ± 4.6	253.2 ± 287	0.843	<0.001	<0.001
Median (IqR)	7.1 (4.6–13.3)	6.4 (4.4–11)	6.4 (4.5–10.6)	236 (6.95–331.8)			
Vitamin B12 (pg/mL)							
Mean ± SD	428.2 ± 252.6	470.8 ± 292	374.2 ± 153.9	324.4 ± 138.2	0.026	0.005	0.284
Median (IqR)	359.0 (280.1–472)	376.5 (300.5–511.8)	347 (259–463)	302.5 (262.2–379)			

Data are expressed as mean ± standard deviation (SD) and median (interquartile range, IQR). AD: Alzheimer’s disease; MCI: mild cognitive impairment; SCD: subjective cognitive decline. Aβ1–40: amyloid-beta 1–40; Aβ1–42: amyloid-beta 1–42; Tau: total tau; Tau181p: phosphorylated tau-181; HbA1c: glycated hemoglobin; HDL-chol.: high-density lipoprotein cholesterol; LDL-chol.: low-density lipoprotein cholesterol.

**Table 2:** Genotype and Allele Frequencies in studied groups.

Variable	Genotype/Allele	AD	MCI	SCD	AD vs. MCI	AD vs. SCD	AD vs. (MCI + SCD)
					χ^2^, (*p*-value)	χ^2^, (*p*-value)	χ^2^, (*p*-value)
APOE genotype							
	E2/E2	1, (1.2%)	1, (3%)	1, (2.7%)	0.47, (0.48)	0.36, (0.54)	0.55, (0.45)
	E2/E3	0	0	0	-	-	-
	E2/E4	3, (3.6%)	1, (3%)	0	0.02, (0.88)	1.35, (0.24)	0.69, (0.40)
	E3/E3	44, (52.4%)	17, (51.5%)	16, (43.25%)	0.007, (0.93)	0.85, (0.35)	0.41, (0.51)
	E3/E4	30, (35.7%)	11, (33.4%)	16, (43.25%)	0.06, (0.80)	0.61, (0.43)	0.13, (0.74)
	E4/E4	6, (7.1)	3, (9.1%)	4, (10.8%)	0.12, (0.72)	0.45, (0.49)	0.40, (0.52)
					0.64, (0.95)	2.91, (0.57)	1.88, (0.75)
APOE allele							
	ε2	5	3	2	0.0001, (0.98)	0.01, (0.88)	0.004, (0.94)
	ε3	118	78	48	2.11, (0.14)	1.06, (0.30)	0.12, (0.72)
	ε4	42	17	24	2.32, (0.12)	1.24, (0.26)	0.12, (0.73)
					2.34, (0.31)	1.24, (0.53)	0.13, (0.93)
	* *p*-Value (HWE)	0.004	0.001	0.02			

* Tri-allelic Exact Test for Hardy–Weinberg Equilibrium (HWE). AD: Alzheimer’s disease; MCI: mild cognitive impairment; SCD: subjective cognitive decline.

## Data Availability

The original contributions presented in this study are included in the article. Further inquiries can be directed to the corresponding author.

## Abbreviations

The following abbreviations are used in this manuscript:

MDP	MDP: mitochondrial-derived peptides
HN	Humanin
AD	Alzheimer’s disease
MCI	Mild Cognitive Impairment
SCD	Subjective Cognitive Decline
mtDNA-CN	Mitochondrial DNA Copy Number
APOE	Apolipoprotein E
Aβ	Amyloid-β
